# Callose Deposition Is Required for Somatic Embryogenesis in Plasmolyzed *Eleutherococcus senticosus* Zygotic Embryos

**DOI:** 10.3390/ijms131114115

**Published:** 2012-10-31

**Authors:** Lei Tao, Yang Yang, Qiuyu Wang, Xiangling You

**Affiliations:** 1 College of Life Sciences, Northeast Forestry University, 26 Hexing Road, Harbin 150040, China; E-Mails: taolei2003-01@163.com (L.T.); wqyll@sina.com (Q.W.); 2 College of Landscape, Northeast Forestry University, 26 Hexing Road, Harbin 150040, China; E-Mail: yangyang8271@163.com

**Keywords:** somatic embryogenesis, plasmolysis, callose deposition

## Abstract

Dynamic changes in callose content, which is deposited as a plant defense response to physiological changes, were analyzed during somatic embryogenesis in *Eleutherococcus senticosus* zygotic embryos plasmolyzed in 1.0 M mannitol. During plasmolysis, callose deposition was clearly observed inside the plasma membrane of zygotic embryo epidermal cells using confocal laser scanning microscopy. The callose content of zygotic embryos gradually increased between 0 and 12 h plasmolysis and remained stable after 24 h plasmolysis. During eight weeks induction of somatic embryogenesis, the callose content of explants plasmolyzed for 12 h was slightly higher than explants plasmolyzed for 6 or 24 h, with the largest differences observed after 6 weeks culture, which coincided with the maximum callose content and highest number of globular somatic embryos. The highest frequency of somatic embryo formation was observed in explants plasmolyzed for 12 h. The somatic embryo induction rate and number of somatic embryos per explant were markedly different in zygotic embryos pretreated with plasmolysis alone (78.0%, 43 embryos per explant) and those pretreated with plasmolysis and the callose synthase inhibitor 2-deoxy-d-glucose (11.5%, 8 embryos per explant). This study indicates that callose production is required for somatic embryogenesis in plasmolyzed explants.

## 1. Introduction

Somatic embryogenesis (SE) has been studied as a model system to understand the physiological, biochemical and genetic events during plant embryo development at a molecular level [1]. The induction of SE in cultured tissues is a multifactorial process and the involvement of plant hormones is the most widely studied aspect to date. Other chemical and physical factors, such as the culture medium nutritional components, light intensity or culture medium form (liquid or solid) have also been studied. Stress treatment of explants at the initial stage of culture or during SE has increasingly been recognized as playing an important role in the induction of SE [2,3]. SE can be induced in apical carrot meristems on medium lacking plant growth regulators (PGRs), by pretreatment with stresses such as heavy metal ions (Co^2+^, Ni^2+^, Zn^2+^ and Cd^2+^) [4], high osmotic stress (high sucrose, mannitol and NaCl contents) [5], or high temperature [6]. Similar stress treatments and exogenous PGRs can induce SE in *Arabidopsis*[7]. Stress is thought to induce the expression of specific genes, which acts as a physiological switch towards the embryogenic state in somatic cells [2,8–10].

The rapid synthesis of large amounts of callose in explants is an important physiological defense reaction to stress. Callose (β-1,3-glucan) is involved in various plant biological processes, and is a component of specialized cell walls or cell wall-associated structures at particular stages of growth and differentiation. Callose plays a very important role in plant developmental processes and also in the response to various biotic and abiotic stresses [11]. Callose deposition has been observed during SE in some species, which is generally initiated by stress pretreatment. For example, Dubois *et al.*[12] induced SE by suspending leaf explants from *Cichorium* plantlets in a solution of glycerin (330 mM) and sucrose (60 mM) for four days, and then transferred the explants to medium supplemented with PGRs. After six days culture, the cytoplasmic content of some epidermal and subepidermal cells was denser and callose deposition was observed, which did not disappear until the formation of a 50-μm diameter proembryo group. The authors suggested that callose deposition was an early marker of SE. Similarly, during SE in *Camellia japonica* and coconut, callose only formed at the onset of cell embryogenic competence in stress-pretreated explants [13]. The role of transient callose deposition during SE is commonly thought to be related to cell wall thickening, information interruption and physiological isolation of cell-to-cell communication [14]. However, callose deposition has not been evaluated in explants during stress pretreatment. Therefore, it is not known if callose deposition at the stress pretreatment stage correlates with callose deposition during SE, or if callose deposition at the stress pretreatment stage influences SE.

*Eletherococcus senticosus* Maxim.is an endangered medicinal plant species in the Araliaceae family. In our previous study, callose deposition was observed in *E. senticosus* zygotic embryos in response to mannitol and sucrose plasmolysis. SE could be induced in these plasmolyzed zygotic embryos after eight weeks culture on PGR-free medium [15]. In the present study, we examined the effects of callose deposition in plasmolyzed explants on SE, and the dynamic changes in callose content during SE.

## 2. Results and Discussion

### 2.1. Callose Content in Zygotic Embryos Plasmolyzed with Mannitol

**Figure 1:**
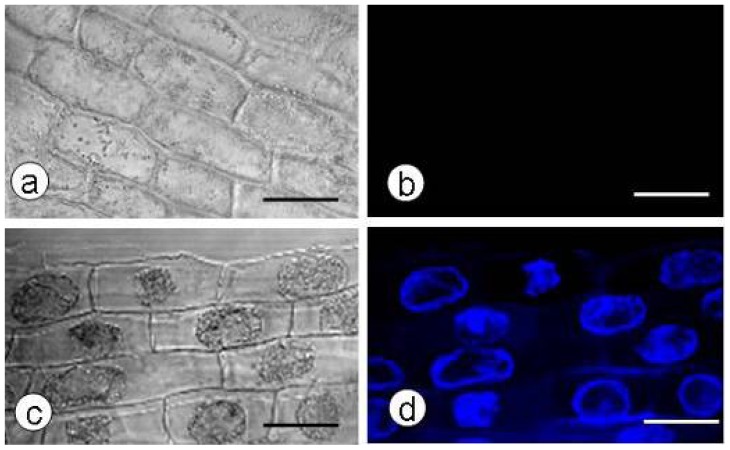
Callose production in the hypocotyl epidermis of plasmolyzed zygotic embryos. (**a**,**b**) Callose deposition was not observed in the hypocotyl epidermis of untreated zygotic embryos. (**c**,**d**) Callose deposition was observed in the cytoplasm of plasmolyzed cells in the hypocotyl epidermis of zygotic embryos pretreated with 1.0 M mannitol for 12 h. Confocal laser scanning microscope bright-field images (**a**,**c**) and aniline blue fluorescent staining of callose (blue; **b**,**d**); scale bar: 50 μm.

Untreated zygotic embryos did not show signs of callose accumulation (Figure 1a,b). Callose deposition was clearly observed inside the plasma membrane of epidermal cells in zygotic embryos plasmolyzed in 1.0 M mannitol for 12 h using CLSM (Figure 1c,d). The callose content increased during 0–12 h plasmolysis, and was not significantly different between 12 h and 24 h plasmolysis (Table 1).

### 2.2. Callose Content in Plasmolyzed Zygotic Embryos during SE

**Figure 2:**
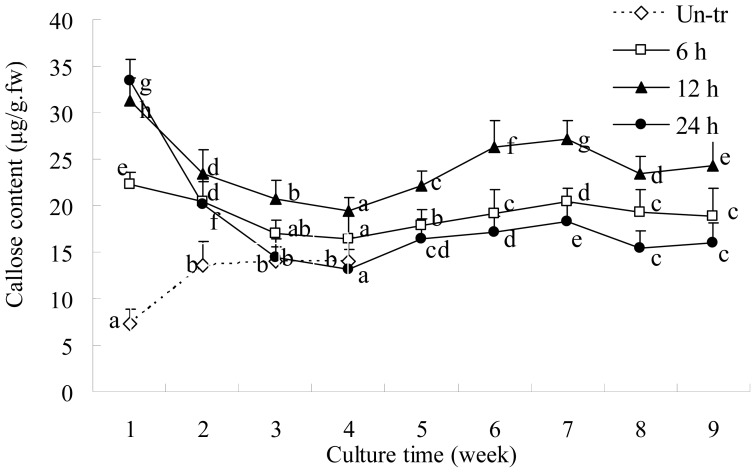
Callose content of zygotic embryos pretreated with 1.0 M mannitol for 0–24 h after 0–8 weeks culture on PGR-free MS. The callose content of non-plasmolyzed zygotic embryos (Un-tr) was only analyzed at four stages, as they developed into plantlets after 2 weeks culture. Data are the mean ± standard error (*n* = 3). Means followed by the same letter within each line were not significantly different at the level of *p* < 0.05, as indicated by Duncan’s multiple range test.

When explants plasmolyzed for 0–24 h were cultured on PGR-free medium, the callose content markedly decreased. During the eight-week induction of SE, the callose content of explants plasmolyzed for different periods of time displayed a similar trend and peaked at 6 weeks (Figure 2); however, the callose content of explants plasmolyzed for 12 h was higher than explants plasmolyzed for 6 or 24 h, and this difference was most obvious at six weeks (Figure 2). The highest frequency of somatic-embryo formation occurred in explants plasmolyzed for 12 h [15], suggesting that the increased callose content was related to SE.

**Figure 3:**
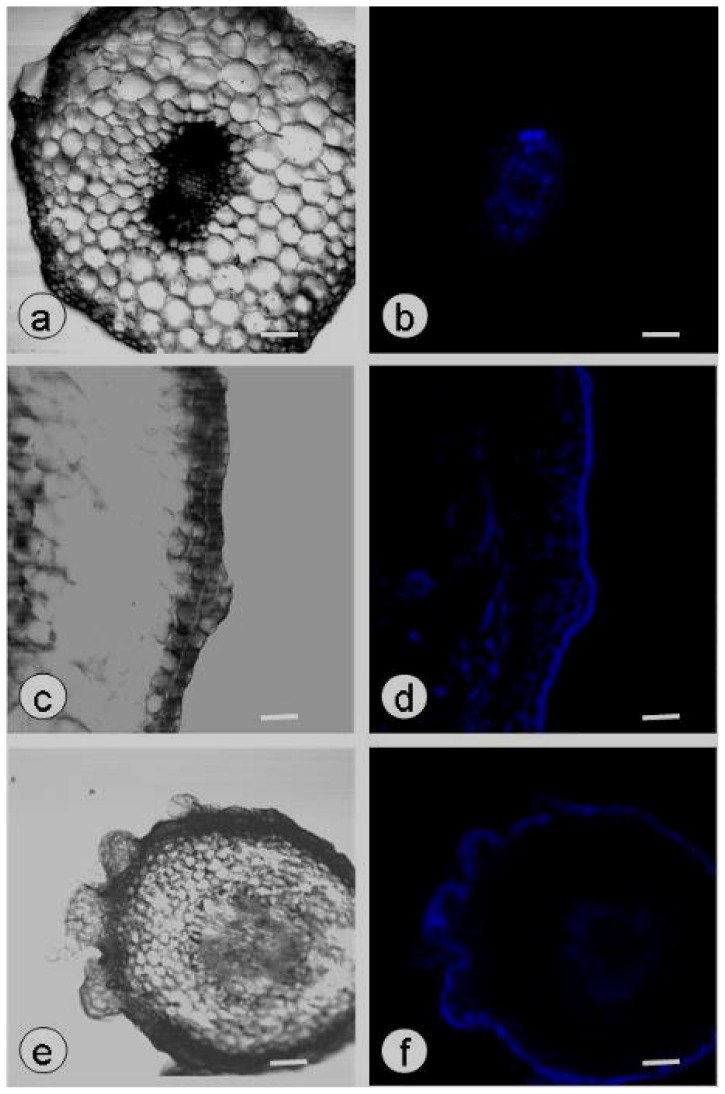
Callose production in the hypocotyls of plasmolyzed zygotic embryos cultured on PGR-free MS. (**a**–**f**) Callose deposition was observed in the vascular tissues of untreated zygotic embryos (**a**,**b**), the epidermis and cortex of plasmolyzed zygotic embryos cultured for four weeks (**c**,**d**) and the globular somatic embryos and vascular tissues of plasmolyzed zygotic embryos cultured for six weeks (**e**,**f**). Confocal laser scanning microscope bright-field images (**a**,**c**,**e**) and aniline blue fluorescent staining of callose (blue; **b**,**d**,**f**); scale bar: 50 μm.

During SE, callose deposition in the hypocotyls of zygotic embryos was assessed by observation of free-hand sections using CLSM. Weak fluorescence was detected in the vascular tissues of untreated zygotic embryos, indicating that only a very small amount of callose was deposited (Figure 3a,b). The epidermis of plasmolyzed zygotic embryos cultured for four weeks had protrusion at localized points due to proliferation of the epidermal cells (Figure 3c), and callose deposition was observed in the epidermal cells and cortical cells (Figure 3c,d). After six weeks culture, somatic embryos had formed on the plasmolyzed explants and callose fluorescence was observed in the epidermis and vascular tissues (Figure 3e,f).

**Figure 4:**
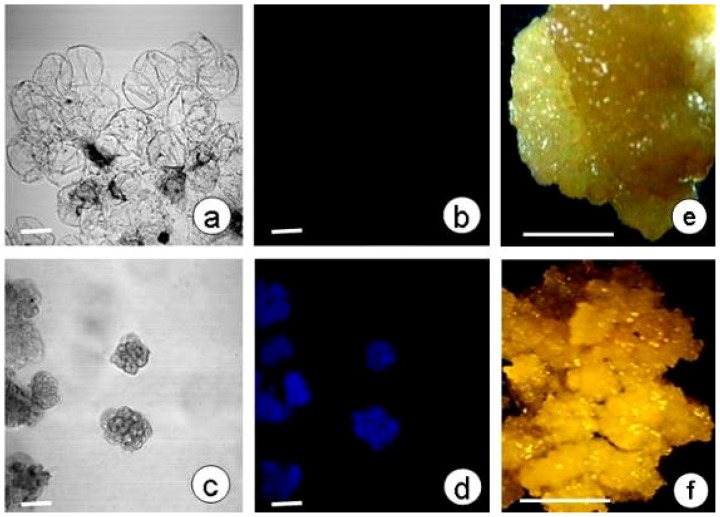
Callose production in non-embryogenic and embryogenic calli. (**a**,**b**) Callose deposition was not observed in non-embryogenic calli. (**c**,**d**) Callose deposition was observed in embryogenic calli. Confocal laser scanning microscope bright-field images (**a**,**c**) and aniline blue fluorescent staining of callose (blue; **b**,**d**); scale bars 100 μm. (**e**,**f**) Light microscope images of a non-embryogenic callus (**e**) and embryogenic callus (**f**); scale bars 0.5 cm.

In order to examine the relationship between callose deposition and SE, callose accumulation was observed in embryogenic (Figure 4f) and non-embryogenic calli (Figure 4e) by CLSM. Callose deposition was apparent in embryogenic calli (Figure 4c,d), but not in non-embryogenic calli (Figure 4a,b), suggesting that SE is related to callose deposition.

### 2.3. DDG-Inhibition of Callose Deposition in Zygotic Embryos

In order to further confirm the effect of callose deposition in plasmolyzed explants during SE, we applied an inhibitor of callose production, DDG, at the same time as plasmolysis treatment. Firstly, we determined the optimal concentration of DDG. At 50–200 μM DDG, the callose content of the explants did not differ significantly, and was considerably higher than the callose level observed in untreated explants (Table 2). The callose content of explants treated with 500–2000 μM DDG was relatively stable and lower than explants treated with 50–200 μM DDG (Table 2). Therefore, we decided that 500 μM was a suitable DDG concentration.

The finding that DDG treatment did not reduce the callose content of plasmolyzed explants to the levels observed in untreated explants may be due to the altered environmental conditions experienced by plasmolyzed explants. When the zygotic embryos were removed from the seed endosperm, they were immersed in solution supplemented with mannitol and DDG, and therefore suffered stress which may have led to some callose deposition. Although a suitable DDG concentration could inhibit callose deposition, it could not fully replicate the original environment of the endosperm.

### 2.4. SE and Callose Deposition after DDG Treatment

**Figure 5:**
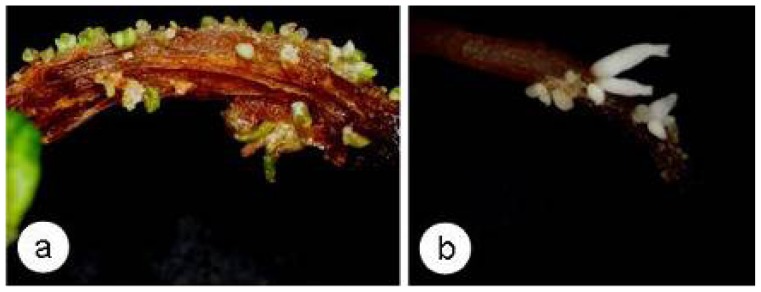
Somatic embryos induced from zygotic embryos pretreated with plasmolysis with 1 M mannitol (**a**) and plasmolysis + 500 μM DDG (**b**) for 12 h after eight-week culture on PGR-free MS medium.

SE was induced in zygotic embryos pretreated with 1.0 M mannitol and 500 μM DDG for 12 h, which were then cultured for eight weeks on PGR-free MS medium (3% sucrose) (Figure 5a,b). AS the development of those somatic embryoes were not synchronous, they were at globular, heart-shaped and torpedo stages after the eight-weeks. The somatic embryo induction rates were markedly different in the explants in the plasmolysis alone and plasmolysis + DDG treatment groups (78.0% and 11.5%, respectively), with 43 and eight somatic embryos formed per explant, respectively. These results indicated that DDG treatment significantly inhibited SE.

**Figure 6:**
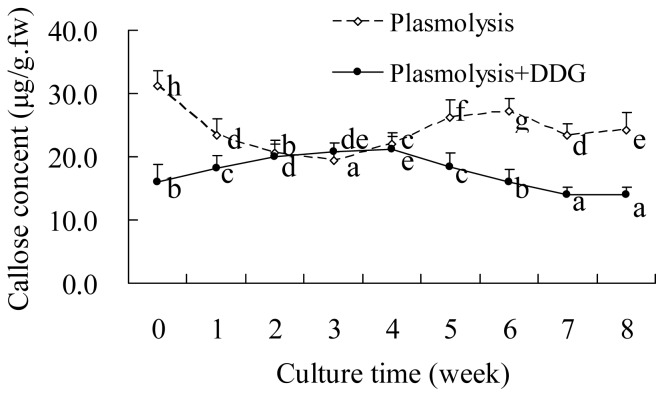
Callose content of zygotic embryos pretreated with 1.0 M mannitol and 500 μM DDG for 12 h after 0–8 weeks culture on PGR-free MS. Data are the mean ± standard error (*n* = 3). Means followed by the same letter within each line were not significantly different at the level of *p* < 0.05, as indicated by Duncan’s multiple range test.

During the eight-week period of SE, the change in the callose content of explants in the plasmolysis + DDG group differed to the plasmolysis only explants (Figure 6). During the first four weeks, the callose content gradually increased in the plasmolysis + DDG pretreated explants, then subsequently decreased (Figure 6), suggesting that changes in the callose content may possibly be related to SE.

This study demonstrates that a significant amount of callose was deposited in the epidermis and vascular tissues of plasmolyzed zygotic embryos. When these embryos were cultured on PGR-free medium, they were unable to develop into seedlings, probably as the vascular tissues were blocked by callose, which may hinder the growth of the root and shoot poles in plasmolyzed zygotic embryos. Therefore, communication between the epidermal cells and cortex was reduced by callose accumulation, which favored somatic embryo formation in epidermal cells. Thus, callose deposition in plasmolyzed zygotic embryos altered the growth of the explants and also the relationship of the epidermis with the cortex and epidermal cells, consequently inducing somatic embryo formation. The callose contents of zygotic embryos plasmolyzed for different periods of time displayed a similar trend during SE, and the callose content remained highest in the zygotic embryos which produced the highest frequency of somatic embryos. Inhibition of callose accumulation reduced the callose content and resulted in the formation of fewer somatic embryos. These results suggest that callose deposition is required for SE, and also that there is a relationship between callose deposition induced by plasmolysis and SE.

## 3. Experimental Section

### 3.1. Callose Deposition in Zygotic Embryos after Plasmolysis and during SE

In our previous study, mature *E. senticosus* zygotic embryos (7 mm long) were stratified in moist sand at 10 °C for 6 months, then plasmolyzed in 1.0 M mannitol for 0–24 h and cultured on PGR-free MS (Murashige and Skoog 1962) medium. The highest frequency of somatic-embryo formation in the plasmolyzed explants was achieved with 12 h pretreatment [15]. In the present study, we analyzed callose deposition in 90 mature zygotic embryos (7 mm long) during plasmolysis in 1.0 M mannitol for 0–24 h, and in 90 mature plasmolyzed zygotic embryos during SE. After plasmolysis, the 180 embryos were rehydrated by gradually decreasing the concentration of osmoticum (mannitol) from 1.0 M to 0.09 M at 5 min intervals per dilution. Ninety embryos were immediately frozen at −80 °C for the callose assay. The remaining embryos were cultured on PGR-free MS medium (3% sucrose) for 8 weeks. At weekly intervals, 100 mg of the embryos were collected and frozen at −80 °C for the callose assay. All media were solidified with 3.0 g/L gellan gum (Gelrite; Kelco Div., Merck & Co., Inc.) and adjusted to pH 5.8 before autoclaving at 121 °C for 15 min. The culture room was maintained at 23 ± 2 °C with a 16h photoperiod and a light intensity of 50 μmol/m^2^/s under cool white fluorescent tubes.

### 3.2. Induction of SE in Plasmolyzed Explants and Inhibition of Callose Deposition

The protein glycosylation inhibitor 2-Deoxy-d-glucose (DDG) inhibits callose production. Zygotic embryos were immersed in 1.0 M mannitol and DDG at eight concentrations (from 0 to 2000 μM) was added to the treatment solution for 12 h. Three hundred milligrams of explants from each treatment were placed in cold storage to assay the callose content. The remaining explants were cultured on PGR-free MS medium (3% sucrose) to induce SE and were sampled at weekly intervals. After eight weeks, the frequency of somatic-embryo formation was scored.

### 3.3. Culture of Embryogenic and Non-Embryogenic Calli

In order to study the relationship between callose deposition and somatic embryogenic, we cheched the callose in embryogenic and non-embryogenic calli. The embryogenic calli were obtained through non-plasmolyzed somatic embryos cultured on MS medium supplemented with 4.4 μM 2,4-dichlorophenoxy acetic acid (2,4-D) for four weeks. The non-embryogenic calli were induced from the hypocotyl segments of somatic embryos cultured on MS medium (3% sucrose) supplemented with 2.0 mg/L naphthylacetic acid (NAA) and 1.0 mg/L 6-benzylaminopurine (BA) for four weeks [16]. Callose accumulation in the calli was observed by confocal laser scanning microscopy (CLSM).

### 3.4. Spectrofluorometric Assay of Callose Content

The callose contents of zygotic embryos during preplasmolysis and SE were assayed using a modification of the methods described by Hirano *et al.*[17]. Briefly, samples (100 mg) were ground to a powder in liquid N^2^ washed three times with 20% (*v*/*v*) ethanol, then 1 mL of 1 M NaOH was added to the powder and the resulting suspension was incubated at 80 °C for 15 min to solubilize the callose. The extract was centrifuged at 10,000 rpm for 5 min and the supernatant was assayed for callose. The callose assay mixture contained 0.2 mL supernatant, 0.4 mL of 0.1% (*w*/*v*) aniline blue, 0.21 mL of 1 M HCl and 0.59 mL of 1 M glycine-NaOH buffer (pH 9.5). Blank controls contained the assay mixture but lacked aniline blue, and the reaction controls lacked supernatant. The reaction mixture was incubated for 20 min at 50 °C, then for 30 min at room temperature to decolorize the aniline blue. The reaction mixture fluorescence was measured in a spectrofluorometer (Cary Eclipse; VARIAN, Palo Alto, CA, USA) at excitation and emission wavelengths of 400 and 510 nm, respectively. Calibration curves were established using fresh solutions of 1,3-β-d-glucan pachyman equivalents (Fluka, Buchs, Switzerland) in 1 M NaOH. From this calibration curve, the amount of callose was expressed as the pachyman equivalents.

### 3.5. Localization of Callose by Confocal Laser Scanning Microscopy

Zygotic embryos plasmolyzed for different periods of time during the induction of SE were fixed with 4% paraformaldehyde in 0.1 M phosphate buffer, rinsed in 0.1 M phosphate buffer and free-hand thin sections (10–12 μm) were cut. The sections were incubated in 0.05% aniline blue in 0.1 M phosphate buffer (pH 7.4) for 5 min. Subsequently, the sections were rinsed with water, dried, and the fluorescence was used to access the callose deposition in an optical section series using the LSM510 META NLO CSLM system (Carl Zeiss Jena, Oberkochen, Germany) using Plan-Neofluar 10× (numerical aperture 0.25) or 20× (numerical aperture 0.75) objectives. The samples were excited at 364 nm using a UV Ar laser (80 mW). For visualization of aniline blue staining, the emission window was set at 535–590 nm with a bandpass emission filter. Excitation at 488 nm was used to obtain bright-field images. Digital images were processed with an AxioCam HR (DXC-950R, Sony) camera using the LSM 5 imaging software [15].

The embryogenic and non-embryogenic calli were respectively fixed with 4% paraformaldehyde in 0.1 M phosphate buffer, rinsed in 0.1 M phosphate buffer. A few calli were prepared on glass slides. For detection of callose, the calli were then incubated in 0.05% aniline blue in 0.1 M phosphate buffer (pH 7.4) for 5 min. Subsequently, the calli were rinsed with water, dried, and the fluorescence was assessed using the above method.

### 3.6. Condition of Tissue Culture and Statistical Analysis

Tissue culture room was maintained in 16/8 h (light/dark) photoperiod with white fluorescent light (80 μmol m^−2^ s^−1^), and 23 ± 2 °C. One-way analysis of variance (ANOVA) using SPSS software (version 17.0) was employed to assess the presence of differences in the callose content of the palsmolyzed explants. Comparisions between the mean values were made using Duncan’s multiple range test (*p* < 0.05).

## 4. Conclusions

In this study, dynamic changes in callose content, which is deposited as a plant defense response to physiological changes, were analyzed during SE in *Eleutherococcus senticosus* zygotic embryos plasmolyzed in 1.0 M mannitol. During plasmolysis, callose deposition was clearly observed inside the plasma membrane of zygotic embryo epidermal cells using confocal laser scanning microscopy. Subsequently, during the induction of SE, the maximum callose content of explants plasmolyzed for 12 h coincided with the highest number of globular somatic embryos. The treatment of the callose synthase inhibitor 2-deoxy-d-glucose obviously reduced the somatic embryo induction rate and number of somatic embryos per explant. These results suggest that callose deposition is required for SE in plasmolyzed explants.

We are currently investigating the genetic basis of plasmolysis- and SE-induced callose production. Biochemical evidence and molecular studies in several plant species have indicated that callose is synthesized by a class of enzymes, termed callose synthases. Twelve genes that encode putative callose synthases (AtCalS1-12) have been identified in the model plant, Arabidopsis thaliana. Some of these genes have been linked to plant growth and development; for example, CalS1, CalS8 and CalS10 are related to biotic and abiotic stress, and CaLS12 is thought to be responsible for pathogen defense. Some putative callose synthases are involved in both biotic and abiotic stress and pathogen defense, including CaLS5, which is thought to be responsible for the formation of the callose wall that separates the microspores of the tetrad and has also been linked to wounding and pathogen defense in a number of plant species [18–20]. We are currently in the process of determining whether the callose generated in response to preplasmolyzing treatment and during SE are produced by the same callose synthase, and some limited data have been obtained. The callose synthase gene, EsCS1, was found through Rt-PCR in zygotic embryos to have increased expression levels when explants were plasmolyzed [21]. The gene will be studied further during SE.

## Figures and Tables

**Table 1:** Callose content of zygotic embryos plasmolyzed with 1.0 M mannitol for 0–24 h.

Plasmolysis time (h)	Callose concentration (μg/g.fw)
Control	6.2 ^d^ ± 1.42
3.0	17.3 ^c^ ± 2.21
6.0	22.2 ^b^ ± 3.33
12.0	30.5 ^a^ ± 3.54
24.0	31.3 ^a^ ± 2.55

Note: Each value is the mean ± somatic embryogenesis (SE) (*n* = 3). Means followed by the same letter within each column are not significantly different at the *p* < 0.05 indicated by a Duncan’s multiple range test.

**Table 2:** Callose content of zygotic embryos pretreated with 1.0 M mannitol and different concentrations of DDG for 12 h.

DDG concentration (μM)	Callose concentration (μg/g.fw)
Untreated	7.1 ^a^ ± 1.32
control	31.3 ^e^ ± 2.13
50	19.5 ^c^ ± 1.51
100	20.3 ^cd^ ± 1.22
200	20.6 ^d^ ± 1.85
500	16.1 ^b^ ± 1.05
1000	15.5 ^b^ ± 1.62
2000	15.8 ^b^ ± 1.44

Note: Untreated zygotic embryos were not pretreated with either plasmolysis or DDG. Each value is the mean ± SE (*n* = 3). Means followed by the same letter within each line were not significantly different at the level of *p* < 0.05, as indicated by Duncan’s multiple range test.

## Abbreviations

SE: somatic embryogenesis
2,4-D: 2,4-dichlorophenoxy acetic acid
BA: 6-benzylaminopurine
CLSM: confocal laser scanning microscopy
DDG: 2-deoxy-d-glucose
NAA: naphthylacetic acid
PGR: plant growth regulator

## References

[1] Zimmerman J.L. (1993). Somatic embryogenesis: a model for early development in higher plants. Plant Cell.

[2] Fehér A., Pasternak T.P., Dudits D. (2003). Transition of somatic plant cells to an embryogenic state. Plant Cell Tissue Org. Cult.

[3] Nolan K.E., Saeed N.A., Rose R.J. (2006). The stress kinase gene MtSK1 in *Medicago. truncatula* with particular reference to somatic embryogenesis. Plant Cell Rep.

[4] Kiyosue T., Takano K., kamada H., Harad H. (1990). Induction of somatic embryogenesis in carrot by heavy metal ions. Can. J. Bot.

[5] Kiyosue T., Satoh S., Kamada H., Harada H. (1993). Somatic embryogenesis in higher plants. J. Plant Res.

[6] Kamada H., Tachikawa Y., Saitou T., Harada H. (1994). Heat stresses induction of carrot somatic embryogenesis. Plant Tissue Cult. Lett.

[7] Iwai M.I., Umehara M., Satoh S., Kamada H. (2003). Stress-induced somatic embryogenesis in vegetative tissues of *Arabidopsis thaliana*. Plant J.

[8] Namasivayam P. (2007). Acquisition of embryogenic competence during somatic embryogenesis. Plant Cell Tissue Org. Cult.

[9] Karami O., Saidi A. (2010). The molecular basis for stress-induced acquisition of somatic embryogesis. Mol. Biol. Rep.

[10] Ma J., He Y.H., Hu Z.Y., Xe W.T., Xia J.X., Guo C.H., Lin S.Q., Cao L., Chen C.J., Wu C.H. (2012). Characterization and expression analysis of AcSERK2, a somatic embryogenesis and stress resistance related gene in pineapple. Gene.

[11] Jacobs A.K., Lipka V., Burton R.A., Panstruga R., Strizhov N., Schulze-lefert P., Fincher G.B. (2003). An Arabidopsis callose synthase, GSL5, is required for wound and papillary callose formation. Plant Cell.

[12] Dubois T., Guedira M., Dubois J., Vasseur J. (1991). Direct somatic embryogenesis in leaves of Cichorium. A histological and S.E.M study of early stages. Protoplasma.

[13] Verdeil J.L., Hocher V., Huet C., Grosdemange F., Escoute J., Ferriere N., Nicole M. (2001). Ultrastructural changes in cocunut calli-associated with the acquisition of embryogenic competence. Ann. Bot.

[14] Grimault V., Helleboid S., Vasseur J., Hilbert J.L. (2007). Co-Localization of β-1,3-Glucanases and callose during somatic embryogenesis in cichorium. Plant Signal. Behav.

[15] You X.L., Yi J.S., Choi Y.E. (2006). Cellular change and callose accumulation in zygotic embryos of *Eleutherococcus senticosus* caused by plasmolyzing pretreatment result in high frequency of single-cell-derived somatic embryogenesis. Protoplasma.

[16] You X.L. (2005). *In Vitro* Mass-Propagation of *Eleutherococcus senticosus* Maxim. Ph.D. Thesis.

[17] Hirano Y., Pannatier E.G., Zimmermann S., Brunner I. (2004). Induction of callose in roots of Norway spruce seedlings after short-term exposure to aluminum. Tree Physiol.

[18] Dong X., Hong Z., Sivaramakrishnan M., Mahfouz M., Verma D.P. (2005). Callose synthase (CalS5) is required for exine formation during microgametogenesis and for pollen viability in *Arabidopsis*. Plant J.

[19] Abercrombie J.M., O’Meara B.C., Moffett A.R., Williams J.H. (2011). Developmental evolution of flowering plant pollen tube cell walls: Callose synthase (CalS) gene expression patterns. EvoDevo.

[20] Hofmann J., Youssef-Banora M., de Almeida-Engler J., Grundler F.M. (2010). The role of callose deposition along plasmodesmata in nematode feeding sites. Mol. Plant Microb. Interact.

[21] Hu X.L., An Y., Xia D.A., You X.L. (2010). Plasmolysis treatment enhances the expression of callose synthase gene in zygotic embryos of *Eleutherococcus senticosus*. J. Forest Res.

